# A systematic review of nonpharmacological interventions to reduce procedural anxiety among patients undergoing radiation therapy for cancer

**DOI:** 10.1002/cam4.6573

**Published:** 2023-10-06

**Authors:** Erin Forbes, Amanda L. Baker, Ben Britton, Kerrie Clover, Eliza Skelton, Lyndell Moore, Tonelle Handley, Sharon Oultram, Christopher Oldmeadow, Alison Gibberd, Kristen McCarter

**Affiliations:** ^1^ School of Medicine and Public Health, College of Health, Medicine and Wellbeing University of Newcastle Callaghan Australia; ^2^ Hunter New England Mental Health Services Newcastle Australia; ^3^ Psycho‐Oncology Service, Department of Consultation Liaison Psychiatry Calvary Mater Newcastle Waratah Australia; ^4^ Flinders Health and Medical Research Institute, College of Medicine and Public Health, Flinders University Bedford Park Australia; ^5^ Department of Radiation Oncology Calvary Mater Newcastle Waratah Australia; ^6^ Data Sciences, Hunter Medical Research Institute New Lambton Australia; ^7^ School of Psychological Sciences, College of Engineering, Science and Environment University of Newcastle Callaghan Australia

**Keywords:** anxiety, oncology, procedural anxiety, procedure‐related anxiety, psycho‐oncology, radiation oncology, radiation therapy, radiotherapy

## Abstract

Procedural anxiety is a concern for a number of patients undergoing radiation therapy. While procedural anxiety is often treated pharmacologically, there is a clinical need for effective alternative strategies for patients who are contraindicated from medication use, and those who prefer not to take unnecessary medications.

**Objectives:**

The primary objective was to assess the efficacy of nonpharmacological interventions delivered to adults with cancer, in the radiation oncology department, just prior to, or during radiation therapy, in reducing levels of self‐reported procedural anxiety. The secondary objectives were to assess the efficacy of these interventions in reducing physiological symptoms of procedural anxiety and anxiety‐related treatment disruptions.

**Design:**

Systematic review.

**Data Sources:**

Electronic databases (MEDLINE, CINAHL, EMBASE, PsycINFO and Cochrane Central Register of Controlled Trials) were searched from inception up until February 2022.

**Inclusion Criteria:**

Population: Adult patients with cancer undergoing external beam radiation therapy. Intervention: Nonpharmacological interventions delivered within the radiation therapy department. Comparison: standard care controls, or standard care plus an alternative intervention. Outcomes: level of self‐reported procedural anxiety (primary), physiological symptoms of anxiety (secondary) and measures of anxiety‐related treatment disruptions (secondary).

**Data Extraction and Analysis:**

Two reviewers independently extracted data. A meta‐analysis was originally planned but deemed not feasible as the studies could not be confidently pooled for meta‐analysis, due to the variability in the interventions, study designs and the generally low number of studies. Therefore, a narrative synthesis is presented.

**Results:**

Screening of 2363 records identified nine studies that met inclusion criteria: six studies of music interventions, two of video‐based patient education and one of aromatherapy. Overall, three studies received a global rating of strong methodological quality and low risk of bias. Three studies reported a significant effect of the intervention on reducing the primary outcome of self‐reported procedural anxiety: two music interventions (both strong methodological quality), and one video‐based patient education (moderate methodological quality). One of the studies (a music intervention) also reported a significant reduction in the secondary outcome of physiological symptoms of procedural anxiety (systolic blood pressure).

**Conclusions:**

The evidence for nonpharmacological interventions delivered to adults with cancer just prior to, or during radiation therapy, in reducing levels of self‐reported procedural anxiety is limited, with very few well‐designed studies. There is a need for interventions for procedural anxiety during radiation therapy to be evaluated through rigorous randomised controlled trials.

## Graphical abstract

The purpose of this review is to summarise the evidence of nonpharmacological interventions targeting procedural anxiety in patients undergoing RT. Specifically, this review will focus on interventions that could feasibly be incorporated into existing procedures within the RT department, offering maximum utility for healthcare providers who manage patient anxiety. There were nine studies that met criteria for inclusion: six studies of music interventions, two of video‐based information‐provision and one of aromatherapy. The evidence for nonpharmacological interventions delivered to adults with cancer, just prior to, or during radiation therapy, in reducing levels of self‐reported procedural anxiety is limited, with very few well‐designed studies.

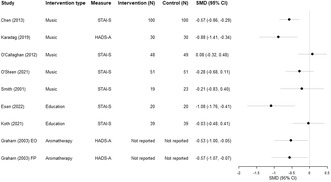

## 1 INTRODUCTION

In 2021, more than 150,000 people were diagnosed with cancer in Australia.^1^ Approximately half of those diagnosed with cancer (48%) were expected to receive radiation therapy (RT),^2^ with more than 74,000 courses of RT delivered in Australia in the 2018–2019 reporting year.^3^ RT is an effective treatment method that can be used as a complete treatment course for cancer, or in combination with other treatment methods (e.g. surgery and/or chemotherapy).^4^ The treatment delivery of external beam RT is noninvasive and painless,^5^ although it does have several side effects, such as fatigue, nausea and skin rashes.^4^ Additionally, many patients experience significant symptoms of anxiety, particularly at the commencement of treatment.^6^

There are many factors that contribute to patient anxiety in oncology settings, including worry about the disease course, concern about side effects, and of course, fear about mortality.^7^ As well as this, many patients experience fear or anxiety relating to medical procedures,^7^ which is referred to as ‘procedural anxiety’. In this paper, we use the term ‘procedural anxiety’ to refer specifically to the acute anxiety experienced in relation to undergoing a healthcare‐related procedure. This is distinct from anxiety related to concerns about what the results of a diagnostic procedure might show, which has been termed ‘scanxiety’.^8^ In some cancer populations (e.g. head and neck cancer), procedural anxiety is seen in as many as 26% of patients.^9^ The fear or anxiety can occur during, or in anticipation of the procedure, and is generally transient.^10^ However, it is associated with acute distress and may result in behavioural disruption such as avoiding or terminating medical procedures.^10^, ^11^ In RT, this is particularly common during the first few treatment sessions,^11^ and persists for some patients through the course of treatment.^12^

Acute anxiety, such as procedural anxiety, is primarily managed in RT settings pharmacologically, with benzodiazepines or nonbenzodiazepine anxiolytics.^7^ However, pharmaceutical management of procedural anxiety presents its own issues. Delays in waiting for a radiation oncologist to prescribe and administer medication, as well as the time waiting for the medication to take effect (up to 30 min)^13^ can be a lengthy process. Additionally, benzodiazepines in particular present problems for longer term use, making them unsuitable for patients experiencing persistent procedural anxiety.^5^ Further, patients who have been given benzodiazepines are advised not to drive while they are affected by the drug, inconveniencing patients and carers. There is also a subset of patients who have contraindications for benzodiazepines, including those with alcohol use disorder, those who are using opioids, as well as elderly patients.^7^, ^13^ Furthermore, many patients report a strong preference to avoid medication where possible.^9^

In addition to pharmacological treatment, studies have investigated nonpharmacological interventions, including music listening,^14^, ^15^, ^16^, ^17^, ^18^, ^19^ education^20^, ^21^ and aromatherapy,^22^ to reduce procedural anxiety in patients undergoing RT. Radiation oncology staff routinely provide informal support to patients experiencing procedural anxiety, although the nature and prevalence of this support has not, to our knowledge, been formally documented. In 2018, Nunns and colleagues^23^ performed a systematic review and meta‐analysis of trials of nonpharmacological interventions aiming to reduce procedural anxiety in paediatric patients undergoing treatment for cancer.^23^ Included in the review were studies trialling hypnosis,^24^, ^25^, ^26^, ^27^, ^28^, ^29^, ^30^ distraction (interactive CD‐ROM,^31^ heated pillow,^32^ listening to music,^33^ virtual reality,^34^ general distraction,^30^, ^34^ games or books^35^ and an interactive device),^36^ cognitive behaviour therapy^27^ and music therapy.^37^ The review reported some promising findings in the studies of hypnosis,^27^, ^28^, ^29^, ^38^ however, cautioned that the studies were primarily conducted by a single research group.

We are not aware of any previous systematic review of nonpharmacological interventions aiming to reduce procedural anxiety among adult oncology patients. This is despite the high prevalence of procedural anxiety, its potential implications (e.g. treatment interruption and patient distress) and the increasing number of studies examining nonpharmacological interventions to address procedural anxiety. A summary of the evidence is needed in order to guide research and clinical practice. The purpose of this review is to summarise the evidence of nonpharmacological interventions targeting procedural anxiety in patients undergoing RT. To provide healthcare providers with maximum utility in managing patient anxiety, this review will focus on interventions that can be feasibly integrated into current procedures within the RT department (i.e. interventions that do not require additional appointments, or specialised external staff). It endeavours to address the needs of all cancer care centres, including those lacking access to psycho‐oncology services as well as those that face substantial wait‐lists.^39^

### 1.1 Objectives

The primary objective of this review was to:

i Assess the efficacy of nonpharmacological interventions delivered to adult patients with cancer, in the radiation oncology department, just prior to, or during RT, in reducing levels of self‐reported procedural anxiety.

The secondary objectives of this review were to assess the efficacy of nonpharmacological interventions delivered to adult patients with cancer just prior to, or during RT, in reducing additional measures of procedural anxiety:

ii Physiological symptoms of procedural anxiety

iii Anxiety‐related treatment completions and duration

## 2 METHODS AND ANALYSIS

This review is registered within the PROSPERO database (registration number CRD42019112941) and is being reported in accordance with guidance provided in the Preferred Reporting Items for Systematic Reviews and Meta‐Analyses (PRISMA) statement.^40^ The methods have been reported in more detail previously.^41^

### 2.1 Inclusion criteria—study characteristics

Due to the lack of clarity and use of the specific term ‘procedural anxiety’ in the literature, for the purposes of this review, studies were deemed to be addressing procedural anxiety if they aimed to: address anxiety; the intervention occurred during, or just prior to RT, and within the RT department; and a standardised and valid measure of anxiety was used (e.g. the State Trait Anxiety Inventory (STAI) State subscale (STAI‐S), which is commonly used to measure procedural anxiety).^23^

#### 2.1.1 Participants

Included studies were those involving adults with cancer currently undergoing, or about to undergo external beam RT, with or without concurrent chemotherapy. Studies involving only paediatric patients (under 18 years) or carers only were excluded. Participants undergoing brachytherapy were also excluded, as the procedure for brachytherapy was considered too different to external beam RT to generalise any findings.

#### 2.1.2 Interventions

The primary criterion for the inclusion of nonpharmacological interventions was feasibility for implementation in real‐world clinical settings. The criteria were based on the clinical expertise of the authorship team (particularly the senior radiation therapist (SO)) and included interventions which could be readily implemented with little notice (as anxiety is often not identified prior to treatment)^11^ and without additional departmental resources. These criteria were applied to focus on scalable interventions with maximum potential for translation into routine clinical practice.

Eligible interventions were those that could be delivered within the department of radiation oncology, at an existing appointment and able to be delivered by any healthcare provider within the RT department (i.e. did not require specialised training, such as psychological interventions or alternative therapies). We excluded interventions that: were delivered by licenced mental health providers; were delivered outside of the RT department; required an additional appointment (which included group interventions); and interventions that required extensive additional training for the healthcare provider to deliver (for example yoga, reiki, hypnotherapy and music therapy that included tailored music delivered by a music therapist). Interventions that exceeded 5 min were excluded, unless they were minimally resource intensive. For example, music listening for 15 min in the waiting room, was included, as participants were able to do this alone or alongside treatment (if music was delivered during treatment sessions). However, an education session delivered by a clinician of the same duration was deemed ‘not able to be delivered in usual appointments’, as this type of intervention requires additional resources from staff.

#### 2.1.3 Comparators

Studies of usual care with no intervention controls, or usual care plus an alternate intervention comparison group(s) were included. Studies without a comparison group were excluded.

#### 2.1.4 Outcomes

Studies using any of the following outcome measures were included in this review. Studies that included any of the secondary outcomes did not need to include any of the primary outcomes to be considered eligible for inclusion.

##### Primary outcome

1. Any validated self‐report measure of anxiety, for example, STAI total score,^42^ STAI‐S,^42^ STAI trait subscale (STAI‐T),^42^ hospital anxiety and depression scale (HADS),^43^ visual analogue scales (VAS)^44^ and the beck anxiety inventory^45^

##### Secondary outcomes

1. Physiological measures of anxiety symptoms, including heart rate, respiratory rate, heart rate variability, electroencephalogram, skin conductance and stress hormones
2. Validated measures of psychological distress, such as the distress thermometer^46^, ^47^ (as an alternative measure of negative emotional state)
3. Measures of anxiety‐related treatment disruptions, for example, treatment completion (or treatment termination due to anxiety) and duration of treatment appointments

#### 2.1.5 Types of studies

Study designs with a comparison group were all eligible for inclusion in the review. Observational studies and qualitative studies were excluded.

### 2.2 Information sources

#### 2.2.1 Electronic databases

The following electronic databases were searched for potentially eligible articles published from inception until February 2022: CINAHL, MEDLINE, EMBASE, PsycINFO and Cochrane Central Register of Controlled trials (CENTRAL). The Medline search strategy (Appendix S1) was adapted to the other databases. An initial search was conducted in February 2019, which was then updated in February 2022.

#### 2.2.2 Other sources

Other sources that were searched included:

1. Reference lists of included articles.
2. A hand search of articles published between 2018 and 2022 in the following relevant journals *Complementary Therapies in Medicine*, *Supportive Care in Cancer*, *PsychoOncology* and *Clinical Journal of Oncology Nursing*.
3. A hand search of conference abstracts from the 2019 and 2021 International PsychoOncology Society conference (IPOS). It should be noted that 2020 and 2021 are considered the ‘two previous years’ consistent with the published protocol.^41^ However, the 2020 conference was cancelled due to COVID‐19 restrictions, and thus for this review we have included the 2019 conference abstracts in place of 2020, to ensure we have searched the abstracts from the two most recent conferences.
4. A grey literature search using Google Scholar (including first 200 records).

### 2.3 Study selection

There was no restriction on the year of publication or language. Studies with no full text available were excluded. Articles that did not report original data (reviews, editorials and opinion articles) were also excluded.

### 2.4 Data extraction

Two authors (EF, and either LM or TH) independently extracted the data using a prepiloted data extraction form, which was based on recommendations by the *Cochrane Handbook for Systematic Reviews of Interventions*.^48^ Discrepancies between the reviewers were discussed until a consensus was reached.

#### 2.4.1 Data items

The following information was extracted:

1. Authors, year and journal
2. Participant inclusion criteria, study design and healthcare setting
3. Patient demographic characteristics
4. Intervention characteristics
5. Comparison group
6. Self‐reported measures of anxiety
7. Physiological measures of anxiety symptoms
8. Validated measures of psychological distress (alternative measure of negative emotional state)
9. Treatment completion/duration

### 2.5 Methodological quality and assessment of bias

Two reviewers (EF, and either LM or TH) independently reviewed the included studies and assessed the methodological quality and risk of bias using the Effective Public Health Practice Project Quality Assessment Tool (EPHPP).^49^, ^50^ The adequacy of six domains were assessed. The EPHPP tool, which is suitable for evaluating randomised and nonrandomised designs (e.g. pre–post and case–control),^49^ has been reported to have both content and construct validity, and acceptable inter‐rater reliability.^50^, ^51^ Regular meetings took place to discuss discrepancies until a consensus was reached.

The two reviewers also reviewed the included studies to assess reporting according to the TiDieR checklist.

### 2.6 Data analysis

Due to the heterogeneity in the intervention types, we did not pool all included studies for a meta‐analysis. We also did not pool studies with the same intervention type because of the small number of available studies and heterogeneity in study designs and outcome measurements. Therefore, findings are reported narratively.

In line with recommendations by the Synthesis Without Meta‐analysis (SWiM) in systematic reviews: reporting guideline^52^ we summarised the findings of both primary and secondary outcomes by intervention type, namely: music (with two subcategories: self‐selected music interventions and predetermined music interventions); video‐based patient education; and aromatherapy.

Results for eligible studies are also reported in a Forest plot (see Figure 2). As the studies included different outcome measures, standardised mean differences (SMDs) were estimated.

### 2.7 Grading the strength of evidence

The overall quality of evidence was assessed using the GRADE (Grades of Recommendation, Assessment, Development and Evaluation) approach,^53^ as recommended by the Cochrane Handbook for Systematic Reviews of Interventions.^48^ The primary outcomes were graded by two authors (EF and TH) as ‘high’, ‘moderate’, ‘low’ or ‘very low’, according to the published definitions. Discrepancies between the reviewers were discussed until a consensus was reached.

## 3 RESULTS

### 3.1 Included studies

**FIGURE 1:**
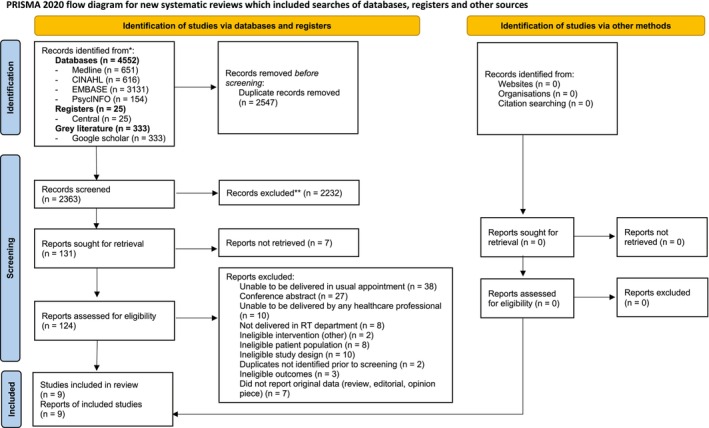
Preferred Reporting Items for Systematic Reviews and Meta‐Analyses flow diagram.

After duplicates were removed, 2363 citations were screened for eligibility. Nine papers reporting results of nine studies met inclusion criteria and are included in this review, with a total sample of 995 participants. The full details of papers identified, screened and included and presented in the Preferred Reporting Items for Systematic Reviews and Meta‐Analyses (PRISMA) flow diagram^54^ (Figure 1).

The primary reasons for exclusion were as follows: interventions that were unable to be delivered in usual appointments (*n* = 38) or unable to be delivered by any healthcare provider (*n* = 10); studies reported in conference abstracts that were not published in a subsequent full report (*n* = 27); ineligible study design (*n* = 10); ineligible patient population (*n* = 8); and not delivered in the RT department (*n* = 8).

#### 3.1.1 Study characteristics

Study characteristics are presented in Table 1. Most studies were conducted in the United States (three studies),^17^, ^19^, ^21^ two studies were conducted in Australia,^16^, ^22^ two in Turkey,^15^, ^20^ one in Taiwan^14^ and one in Italy.^18^ Included papers were published between 2001 and 2022.

**TABLE 1:** Study characteristics.

Study (Country)	Design	Dates	Setting	Aim	Inclusion criteria	Patients (n)	Mean age in years (SD)	Gender (n, % male)	Tumour site/stage *n* (%)
Music interventions									
Chen^14^ (Taiwan)	Quasi‐experimental (randomised design by simple random allocation, i.e. every other patient)	1st April 2011–31st October 2011	Radiation Oncology Department of Far Eastern Memorial Hospital (Taipei)	To investigate effects of music intervention on reducing pre‐RT anxiety in oncology patients	Patients who were as follows: ‐ scheduled to receive a treatment protocol of RT lasting about 5 weeks or more ‐ who were at least 18 years old ‐ had sufficient literacy to respond to a written questionnaire	200	Intervention group: 55.06 (13.50 SD) Control group: 55.66 (11.41 SD)	121 (60.5%) male	Site^a^ Head and neck—67 (33.5%) Gynaecological—23 (11.5%) Breast—38 (19.0%) Digestive tract—37 (18.5%) Lung—12 (6.0%) Prostate ‐ 18 (9.0%) Stage Stage I—35 (17.5%) Stage II—45 (22.5%) Stage III—71 (35.5%) Stage IV—49 (24.5%)
Karadag^15^ (Turkey)	Randomised controlled trial	1st November 2017–October 2018 + 5 weeks	Radiation oncology outpatient clinic of Dokuz Eylul University Medical Faculty Hospital	To examine the effect of a music listening intervention applied during RT on the anxiety and comfort level experienced by women with early‐stage breast cancer	Patients who were as follows: ‐ 18 years and over ‐ diagnosed with breast cancer, stage I or II ‐ having no hearing‐speech problem ‐ receiving no psychiatric treatment (taking no antianxiety/antidepressant drugs) ‐ not in the terminal stage ‐ had cancer in the right breast (because breath‐holding technique is administered to patients with left‐sided breast cancer, they cannot listen to music during the application) ‐ volunteering to participate in the study	60	59.40 (13.28 SD)	60 (100%) female	Site Breast cancer—60 (100%) Stage I—35 (58.3%) II—25 (41.7%)
O'Callaghan^16^ (Australia)	The triangulation mixed method convergence model design comprised a single centre, nonblinded parallel group, randomised controlled trial plus subjective data collection	1st June 2006–1st July 2009	Peter McCallum Cancer Centre	To examine whether patients' use of self‐selected music while undergoing first RT treatment reduces anxiety, and how patients describe their first RT experience with or without self‐selected music	Patients who were as follows: outpatients with a cancer diagnosis at any site ‐ receiving treatment with curative intent ‐ 18 years or older ‐ had received no prior external beam RT	100	Intervention group: 57 (14.2 SD) Control group: 58 (12.7 SD)	59 (59%) male	Site Prostate—42 (42.0%) Cervix—10 (10.0%) Endometrium ‐ 9 (9.0%) Breast—7 (7.0%) Lung—5 (5.0%) Other—27 (27.0%) Stage Staging not reported
O'Steen^17^ (United States)	Prospective randomised trial	1st January 2018–31st December 2019	Department of Radiation Oncology at the University of Florida in Gainesville, Florida	To evaluate the influence of genre‐based music chosen by the participant on anxiety during the first RT treatment session	Patients who were as follows: ‐ Women treated for cancer with RT ‐ ≤18 years of age	102	Median age (range) 62 (32–92)	102 (100%) female	Site Breast—54 (52.9%) CNS—10 (9.8%) GI—5 (4.9%) Head & neck—12 (11.8%) Lung—11 (10.8) Lymphoma—1 (1.0%) Other—9 (8.8%) Stage 0—10 (9.8%) I—35 (34.3%) II—9 (8.8%) III—13 (12.7%) IV—8 (7.8%) Recurrent—27 (26.5%)
Raglio^18^ (Italy)	Randomised controlled pilot study	Not reported	Not reported	To verify if the perception of anxiety and stress in cancer patients undergoing RT is influenced by music listening	Patients who were as follows: ‐ diagnosed with breast cancer ‐ candidates for postoperative curative RT ‐ undergoing RT for the first time in their life	60	Median age (range) Intervention group: 58 (55–67) Control group: 59 (51–68)	60 (100%) female	Site Breast—60 (100%) Stage Staging not reported
Smith^19^ (United States)	Experimental, longitudinal, random assignment to music or no music therapy	Not reported	Radiation Oncology Center at a Department of Veterans Affairs Hospital	To (a) determine whether patients' level of anxiety changes during RT (b) determine whether music moderates the anxiety experienced	Patients who were as follows: ‐ expected to receive at least 5 weeks of RT ‐ at least 18 years of age ‐ able to read and understand English	42	62.8 (39–80 range)	42 (100%) male	Site Prostate—24 (57.1%) Lung—6 (14.3%) Head and neck—4 (9.5%) Colorectal—4 (9.5%) Squamous cell skin—2 (4.8%) Stomach—1 (2.4%) Melanoma—1 (2.4%) Stage Staging not reported
Video‐based patient education									
Esen^20^ (Turkey)	Patients were divided into 2 groups. A simple randomisation method was used to randomise patients by assigning an odd or even number to the patients	1st December 2019 – 1st March 2020	Not reported	To assess the impact of video‐based education on patient anxiety and patients' compliance with the treatment	Patients who were as follows: ‐ receiving stereotactic radiosurgery (SRS) or stereotactic body RT (SBRT) ‐ speaking Turkish, ‐ having the capability of reading, understanding, and responding to the questionnaire in a meaningful way ‐ older than 18 years	40	57 (13 SD)	21 (53%) male	Site Head and neck (including brain lesions)—29 (72.5%) Vertebral metastases—8 (20%) Prostate—1 (2.5%) Abdomen—2 (5%) Stage Staging not reported
Koth^21^ (United States)	Randomised controlled trial	1st March 2016–1st July 2019	Not clear	To characterise the effect of an informational video viewed by patients with head and neck malignancies after their initial radiation oncology consultation to reduce multiple aspects of treatment‐related anxiety	Patients who were as follows: ‐ diagnosed with a head and neck malignancy ‐ 19 years or older	78	62.7 (11.09 SD)	64 (82%) male	Site Oropharynx—30 (38.5%) Oral cavity—15 (19.2%) Nasopharynx—4 (5.1%) Larynx and hypopharynx—12 (15.4%) Salivary gland—6 (7.7%) Skin—4 (5.1%) Thyroid—3 (3.8%) Other—4 (5.1%) Stage 0—3 (3.8%) I—24 (30.8%) II—31 (39.7%) III—15 (19.2%) IV—5 (6.4%)
Aromatherapy									
Graham^22^ (Australia)	Placebo‐controlled double‐blind randomised trial	Not reported	Not clear	To determine whether the inhalation of aromatherapy during RT reduces anxiety	Patients who were as follows: ‐ prescribed a course of eight or more fractions of RT	313	65 (14.25 SD)	162 (52%) male	Site Thoracic—29% Abdominal or pelvic—28% Breast—20% Head and neck—19% Multiple or skin sites—4% Stage Staging included in analysis, but not reported in descriptive information

^a^ Total equals 195. Missing data not explained in the original publication.

#### 3.1.2 Participants

Of the included studies, five included patients with any type of cancer who were scheduled to receive a course of RT,^14^, ^16^, ^17^, ^20^, ^22^ two studies were constrained to patients with breast cancer,^15^, ^18^ one included patients with head and neck malignancies^21^ and one included patients with pelvic or abdominal malignancies.^19^ Participant details including age and sex are included in Table 1.

#### 3.1.3 Interventions

Three types of intervention were identified in the included studies: music (*n* = 6)^14^, ^15^, ^16^, ^17^, ^18^, ^19^; video‐based education (*n* = 2)^20^, ^21^; and aromatherapy (*n* = 1).^22^ A summary of intervention characteristics is included in Table 2.

**TABLE 2:** Intervention characteristics.

Study	Intervention description	Delivery (when)	Delivery (where)	Timing	Comparison condition(s)
Music interventions					
Chen^14^	Slow‐paced, soft, melodic music at low volume with consistent tempo and dynamics and an average 60–80 beats per minute Subjects chose their own favourite music tracks from a selection of old songs in Mandarin, Mandarin pop, traditional Taiwanese songs, Western music (country and western), and classical music (e.g., chamber music with string instruments)	Patients decided individually on which day of their RT schedules their music intervention should be conducted	In waiting room prior to treatment (via headphones)	15 mins	Same procedure but without providing music therapy Patients were instructed to sit comfortably on the radiology waiting room couch (a separate room from the music therapy group but similar facility) and to rest for 15 min
Karadag^15^	Patients in the intervention group listened to a piece chosen by a music and rhythm expert (as relaxing). The piece chosen was Bach's 19 Trio sonatas in which James Galway plays the flute	5 weeks	Treatment bunker during treatment delivery (via headphones)	Duration of treatment session	Patients in the control group received no music listening intervention
O'Callaghan^16^	Participants had received orientation to the RT service at the treatment planning stage Intervention group participants were telephoned and reminded to bring music to their first treatment. Music was heard without headphones at (reasonable) participant desired volume	One‐off treatment (initial RT session)	Treatment bunker during treatment delivery (via speakers)	Duration of first treatment	No music
O'Steen^17^	Patients selected their preferred genre of music from a web‐based application with a directory of pre‐recorded music	One‐off treatment (initial RT session)	Treatment bunker during treatment delivery (via speakers)	Duration of first treatment	No music or other audio in the treatment room
Raglio^18^	The intervention group listened to music produced by Melomics‐Health^10^ (15 min, five songs of about 3 min each). The music proposed (based on relaxing/deactivating music model) was designed to reduce the state of anxiety and stress given by RT. The musical content was designed to act as a psychological deactivator and to be relaxing. All parameters and structures maintained low levels and a consistent course. The music was based on a regular pattern, a monody with a reduced musical density; the time was unchanged and there were no significant dynamic and tonal variations	Before the simulation and the first 5 RT sessions	The listening experience took place in a quiet room inside the RT service where the patients were alone (via headphones)	15 min	The no‐music group did not perform any type of musical listening
Smith^19^	Patients were given a choice of various categories of music, including rock and roll, big band, country and western, classical, easy listening, Spanish and religious. Patients were allowed to select a tape from the category of their choice. Once the choice was made, they were asked to continue to choose a tape from that category for the duration of the study. Four to six tapes were available in each category	During their RT simulation appointment and also during their daily RT sessions	Treatment bunker during treatment delivery (via headphones)	Duration of planned course of RT	No music plus standard care delivered within the department. Standard care provided to all study participants included orientation to the department, procedures and individualised patient education
Video‐based patient education					
Esen^20^	One‐to‐one information session Two educational videos were prepared per SRS/SBRT devices (Cyberknife*®* or Novalis*®*) The video presentations began with a RT simulation process with explanations about reasons for immobilisers' usage and how the patient will feel with immobilisers (etc. when her/his face is covered up with a thermoplastic mask), then treatment areas, movements of RT device during the treatment and brief moments of treatment were displayed in video presentations so that patients had an idea of what to expect	Before treatment	Not reported	2–3 min	One‐to‐one information session only
Koth^21^	Standard face‐to‐face education with treating physician A 5‐min educational video was created that portrayed the creation of a thermoplastic mask, CT simulation, radiation planning and the delivery of RT. The video was narrated and depicted the actor describing his experiences with each part of treatment planning and mock delivery. The Virtual Environment Radiotherapy Training system was used to show details of treatment specific to patients with head and neck malignancies. Cross‐sectional anatomy of the head and neck region also was added to emphasise the importance of immobilisation and precision	One‐off participation—after their initial consultation with the managing radiation oncologist	Not reported	5 min	Standard face‐to‐face education with treating physician
Aromatherapy					
Graham^22^	Patients wore a necklace with a plastic‐backed paper bib (such as worn at the dentist), donned before treatment each day and removed after exiting the treatment bunker Three drops of oil were applied to the bib The essential oils were lavender, bergamot and cedarwood in a ratio of 2:1:1	Each day of treatment	Treatment bunker during treatment delivery	15–20 min	Participants in the fragrant placebo group received the same procedure, with fractionated oils (2:1ratio) applied to the necklace Participants in the nonfragrant placebo group received the same procedure, with carrier oil only, applied to the necklace

##### Music

The music interventions could be broadly divided into those using self‐selected (participant chosen) music (*n* = 4)^14^, ^16^, ^17^, ^19^ and those using predetermined (investigator chosen) music selection (*n* = 2).^15^, ^18^ Interventions also varied in whether the music intervention was delivered prior to (*n* = 2)^14^, ^18^ or during (*n* = 4)^15^, ^16^, ^17^, ^19^ RT; over one session (*n* = 3)^14^, ^16^, ^17^ or many (*n* = 3)^15^, ^18^, ^19^; and via headphones (*n* = 4)^14^, ^15^, ^18^, ^19^ or speakers (*n* = 2).^16^, ^17^

###### Self‐selected music interventions

Chen et al^14^ conducted a quasi‐experimental study (*n* = 200). Participants randomised to the music group were given an opportunity to choose from a selection of old songs (soft, slow‐paced melodic music, 60–80 beats per minute), and then instructed to sit in the waiting room prior to treatment and listen to the selected songs through headphones, for 15 min. Participants in the control group were instructed to sit comfortably on the radiology waiting room couch (a separate room from the music therapy group but similar facility) and to rest for 15 min. The study found a significantly greater reduction (*p* < 0.001) in self‐reported anxiety (mean decrease of 7.19 on the STAI‐S) in the music group participants when compared to the control group (mean score decreased by 1.04) after the intervention with a medium effect size of 0.52. The findings from this study also support a clinically meaningful reduction in procedural anxiety (−7.19 decrease on the STAI‐S) for those in the music group, with the mean anxiety score (35.44) in the normal range (< 40 on the STAI‐S^55^) following exposure to the intervention. A much smaller reduction was seen for the control group (1.04) which remained over the cut off for clinically significant anxiety at follow‐up (40.99). Additionally, the study also reported a significant reduction in systolic blood pressure in the music group (decrease of 5.69 mmHg) compared to the control group (decrease of 0.67 mmHg) with a small effect size of 0.30.

A further three studies trialled music interventions that involved some level of music self‐selection. However, in contrast to Chen and colleagues,^14^ all three interventions were delivered in the RT treatment bunker, during treatment delivery. Two of the three studies^16^, ^17^ conducted randomised trials of self‐selected music during the first RT session only. In both trials the music was delivered via the speakers within the treatment bunker. In the study conducted by O'Steen and colleagues^17^ (*N* = 102), participants randomised to the intervention group selected their preferred genre of music from a directory of prerecorded music, while intervention participants in the study conducted by O'Callaghan and colleagues^16^ (*N* = 100) were asked to bring their own music. The control groups in both trials received no intervention. O'Steen and colleagues^17^ reported that while there was a 16% reduction in the music group compared to a 10% reduction in self‐reported anxiety in the control group, the difference between groups was not significant. Similarly, O'Callaghan reported no significant difference in anxiety reduction between the groups, however, the authors reported that participants in the music group indicated a preference for music at future RT appointments statistically significantly more often than control participants. The third study, conducted by Smith and colleagues^19^ (experimental, longitudinal, random assignment trial *N* = 42) trialled a music listening intervention delivered during the RT simulation appointment, as well as daily treatment sessions. Participants in the experimental group listened to music of their choice from a selection of music provided by the researchers. Participants in the control group did not listen to music and received the standard care of the department. In contrast to the trials conducted by O'Steen^17^ and O'Callaghan,^16^ the music listening intervention was delivered to participants through headphones. The study found no significant effect on anxiety in either group.

###### Predetermined music interventions

The two trials of predetermined music selection were conducted by Karadag and colleagues,^15^ (randomised controlled trial, *N* = 60) and Raglio and colleagues^18^ (randomised controlled pilot study, *N* = 60). In the study conducted by Karadag and colleagues,^15^ intervention participants received an MP3 player with headphones to listen to 19 Trio Sonatas, a piece composed by Bach, with a duration of 20–40 min, during each radiation treatment for a period of 5 weeks. Participants randomised to the control group received no intervention. The study found a statistically significant reduction in anxiety (*p* = <0.001) in the music group (mean score decreased by 2.6 on the HADS Anxiety subscale, HADS‐A) compared with the control group (mean score increased by 1.0) after the intervention (at 5 weeks) with a moderate effect size of 0.58. The findings also support a clinically meaningful reduction in anxiety of 2.6 on the primary outcome measure (HADS‐A), a reduction that exceeds the minimum clinically important difference of 1.7.^56^ Additionally, the mean score for those in the intervention group was no longer in the clinical range (7 or more^57^) following the exposure to the intervention (5.20), compared to those in the control group who remained in the clinical range (8.56).

Raglio and colleagues^18^ trialled two different music interventions: a piece produced by Melomics‐Health (which produces music for therapeutic purposes), and the other group received individualised music listening. A third group (control) received no music intervention. However, the individualised music listening intervention was deemed ineligible for this review due to the involvement of a music therapist, who worked with participants to build their personalised playlist. These data were excluded from our review, but the Melomics‐Health intervention (and control group) data are reported here, as that part of the study meets inclusion criteria. Participants randomised to the Melomics‐Health group were instructed to sit in a quiet room in the RT department prior to RT simulation in the first 5 treatment sessions, where they listened to 15 min of music through headphones. The study found no difference between the Melomics‐Health group and the no music control group on self‐reported anxiety.

##### Video‐based patient education

Two randomised trials examined the effect of video‐based patient education on patients' procedural anxiety. Esen and colleagues^20^ (*N* = 40) trialled a 2–3 min video showing patients what to expect in treatment. The video described the simulation process with explanations about the reason for immobilisers, how the patient may feel and the movements of the treatment machine. Similarly, Koth and colleagues^21^ (*n* = 78) trialled a 5 min educational video with similar content. The video included a demonstration of making the thermoplastic mask, CT simulation, radiation planning and the delivery of radiation treatments, including undergoing a mock treatment delivery and explaining the experience. The control groups in both trials received standard education. Esen and colleagues^20^ found a significant reduction (*p* < 0.001) in anxiety after viewing the video‐based patient education, and after the first treatment session when compared to the control group (mean difference of 10 points on the STAI‐S). Koth and colleagues^21^ reported a significant reduction in one individual survey item of the STAI (adults short form Y‐1) (‘I am worried’) in the intervention group, after viewing the education material. However, there was no significant difference in the total mean anxiety score between the two groups.

##### Aromatherapy

Graham et al^22^ conducted a placebo‐controlled double‐blind randomised trial (*N* = 313) to determine whether aromatherapy reduced anxiety during radiation therapy. Participants were randomised to receive a fragrant placebo, a nonfragrant placebo or pure essential oils (lavender, bergamot and cedarwood). Three drops of oil were applied to a paper bib that participants wore for the duration of their treatment each day. The study found a significant reduction in anxiety (*p* = 0.04) in the nonfragrant placebo group compared to the essential oils and the fragrant placebo (odds ratios 2.8) and concluded that aromatherapy did not reduce anxiety.

#### 3.1.4 Provider

Two interventions were delivered by a member of the research team (*n* = 2)^14^, ^22^; however, most studies did not report who delivered the intervention (*n* = 7).^15^, ^16^, ^17^, ^18^, ^19^, ^20^, ^21^

#### 3.1.5 Outcomes

**FIGURE 2:**
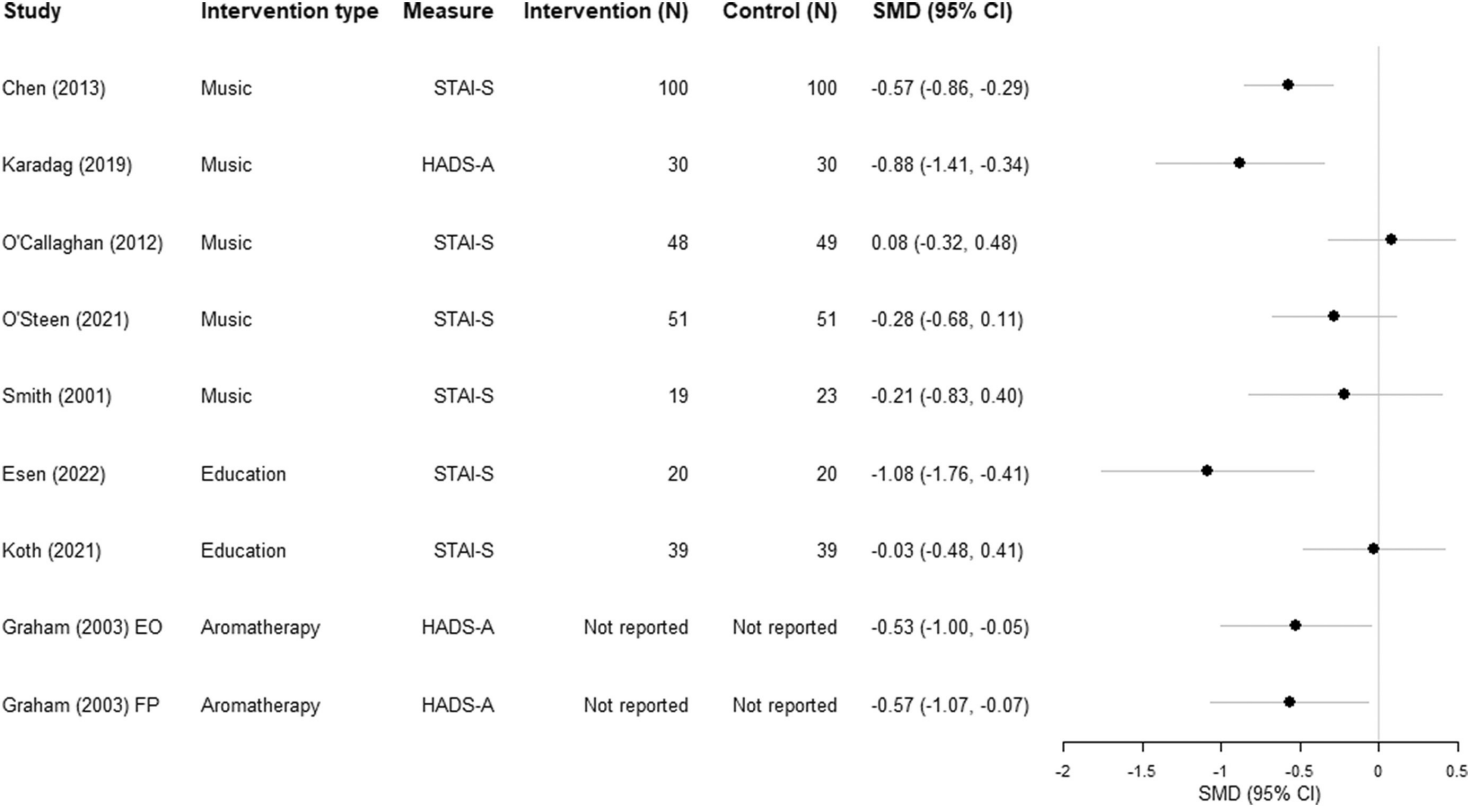
Forest plot of standardised mean differences and description of methodology. SMD, standardised mean difference; CI, confidence interval; EO, essential oil; FP, Fragrant placebo; STAI‐S, Spielberger State–Trait Anxiety Inventory; HADS‐A, Hospital Anxiety and Depression Scale‐Anxiety. Participant numbers for O'Callaghan (2012) are lower than reported in Table 1 as three patients withdrew prior to initial radiation therapy. Graham (2003) included 313 patients but did not report how many were randomised to each group.

Primary and secondary outcomes are presented in Table 3, with summary statistics presented in Table 4. A forest plot of the standardised mean differences is presented in Figure 2. Standardised mean differences (SMDs) were estimated for eight of the nine eligible studies. One study^18^ was not included as there was insufficient information reported to estimate a SMD.

**TABLE 3:** Study outcomes.

Study	Outcome	Results
Music interventions		
Chen^14^	Primary outcome: Mean change in anxiety score from pretest (immediately prior to intervention), and posttest (immediately after intervention, delivered on the same day) Outcome measure: State–Trait Anxiety Inventory—State and Trait subscales (STAI‐S and STAI‐T)^11^, ^12^	Statistical analysis: Independent two‐sample *t* tests. STAI‐S Intervention group: Mean decrease of 7.19 (± 0.94) Control group: Mean decrease of 1.04 (± 0.41) *p* value = <0.001 STAI‐T Intervention group: Mean decrease of 2.77 (± 0.66) Control group: Mean decrease of 1.13 (± 0.42) *p* value = 0.036
	Secondary outcome: Mean change of vital signs from pretest (immediately prior to intervention), and posttest (immediately after intervention, delivered on the same day) Outcome measure: Heart rate, respiratory rate, blood pressure and fingertip oxygen saturation	Statistical analysis: Independent two‐sample *t* tests Heart rate Intervention group: Mean decrease of 4.40 (± 0.77 beat/min) Control group: Mean decrease of 3.28 (± 1.10 beat/min) *p* value = 0.405 Respiration rate Intervention group: Mean decrease of 0.65 (±0.13 breaths/min) Control group: Mean decrease of 0.46 (±0.14 breaths/min) *p* value = 0.319. Systolic pressure Intervention group: Mean decrease of 5.69 (±0.41 mmHg) Control group: Mean decrease of 0.67 (±1.29 mmHg) *p* value = 0.009. Diastolic pressure Intervention group: Mean decrease of 1.71 (±0.89 mmHg) Control group: Mean decrease of 1.41 (±0.86 mmHg) *p* value = 0.809. Fingertip oxygen saturation Intervention group: Mean decrease of 0.01 (±0.13%) Control group: Mean decrease of 0.06 (±0.13%) *p* value = 0.779.
Karadag^15^	Primary outcome: pretest (first day of treatment) and posttest (last day of treatment, week 5) Outcome measure: Hospital Anxiety and Depression Scale (anxiety) (HADSA)^13^	Statistical analysis: ANCOVA HADS‐A Intervention: Mean decrease of 2.60 (2.83 SD) Control: Mean increase of 1.00 (3.30 SD) *p* value = 0.000
O'Callaghan^16^	Primary outcome: Mean change in anxiety score from pre‐RT (immediately prior to the first treatment session) to post‐RT (immediately after the first treatment session). Outcome measure: State–Trait Anxiety Inventory—State and Trait subscales (STAI‐S and STAI‐T) ^11^, ^12^	Statistical analysis: ANCOVA STAI‐S Intervention: Mean decrease of 4 (9.6 SD) Control: Mean decrease of 5 (7.0 SD) *p* value: 0.45 STAI‐T Only baseline STAI‐T scores reported.
O'Steen^17^	Primary outcome: percent change in mean anxiety score from pretreatment (within 2 h prior to first RT session) and post‐treatment (within an hour after their first RT session). Outcome measure: State–Trait Anxiety Inventory—State and Trait subscales (STAI‐S and STAI‐T)^11^, ^12^	Statistical analysis: Independent *t*‐test STAI total (both State and Trait subscales) Intervention: 16% reduction in STAI score Control: 10% reduction in STAI score *p* value = 0.2197 Intervention: 33% change from high (≥40) to low anxiety. Control: 22% change from high (≥40) to low anxiety. *p* value = 0.6363
	Secondary outcome: percent change in mean anxiety score from pretreatment (within 2 h prior to first RT session) and post‐treatment (within an hour after their first RT session). Outcome measure: Symptom Distress Thermometer (SDT)^14^	Statistical analysis: Independent t‐test SDT Intervention: 13% reduction in SDT score Control: 2% increase in SDT score *p* value = 0.3298 Intervention: 17% change from high (≥4) to low anxiety. Control: 13% change from high (≥4) to low anxiety. *p* value = 0.8567
Raglio^18^	Primary outcome: change in proportion of subjects with an outcome score below the critical value (≤ 40), measured at baseline (T0), at the end of treatments (T1) and at follow‐up (T2, 2 weeks after the fifth session of RT) Outcome measure: State–Trait Anxiety Inventory—State and Trait subscales (STAI‐S and STAI‐T)^11^, ^12^	Statistical analysis: chi‐square test or Fisher exact test STAI‐T—Proportion of patients with a STAI‐T score below the critical value (40) *End of treatments* Intervention: 5% increase in patients with a STAI‐T score below the critical value (40) Control: No change *Follow‐up* Intervention: 5% increase in patients with a STAI score below the critical value Control: 5.26% increase in patients with a STAI score below the critical value STAI‐S—Proportion of patients with a STAI‐S score below the critical value *End of treatments* Intervention: 5% increase of patients with a STAI score below the critical value Control: 15.79% increase of patients with a STAI score below the critical value *Follow up* Intervention: 5% increase of patients with a STAI score below the critical value Control: 5.26% increase of patients with a STAI score below the critical value
	Secondary outcome: change in proportion of subjects with an outcome score below the critical value (≤ 35), measured at baseline (T0), at the end of treatments (T1) and at follow‐up (T2, 2 weeks after the fifth session of RT) Outcome measure: Psychological Distress Inventory (PDI)^15^	Statistical analysis: chi‐square test or Fisher exact test PDI—Proportion of patients with a Psychological Distress Inventory (PDI) score below the critical value (35) *End of treatments* Intervention: 16.67% increase of patients with a STAI score below the critical value Control: 18.42% increase of patients with a STAI score below the critical value *Follow up* Intervention: No change Control: 9.12% decrease (from treatment 1) of patients with a STAI score below the critical value
Smith^19^	Primary outcome: Mean change in state anxiety from baseline (time of evaluation) pretreatment (postsimulation), during treatment (at the end of the first week of treatment) and post‐treatment (at the end of the third week). Outcome measure: State–Trait Anxiety Inventory—State subscale (STAI‐S)^11^, ^12^	Statistical analysis: Mixed‐design, two‐way ANOVA STAI‐S Intervention: Mean decrease of 5.5 Control: Mean decrease of 3.1 *p* value = 0.763
	Secondary outcome: Mean change in trait anxiety from baseline (time of evaluation) pretreatment (post simulation), during treatment (at the end of the first week of treatment) and post‐treatment (at the end of the third week). Outcome measure: State–Trait Anxiety Inventory‐ Trait subscale (STAI‐T)^11^, ^12^	Statistical analysis: Mixed‐design, two‐way ANOVA STAI‐T Intervention: Mean decrease of 2.2 Control: Mean decrease of 2.3 *p* value = 0.678
Video‐based patient education		
Esen^20^	Primary outcome: Change in anxiety scores before information sessions, after information sessions and after treatment (immediately after treatment delivered on the same day as the intervention) Outcome measure: State–Trait Anxiety Inventory—State and Trait subscales (STAI‐S and STAI‐T)^11^, ^12^	Statistical analysis: Friedman test and Wilcoxon test with Bonferroni correction. STAI‐S (Medians and IQR) *Before education* Intervention group: 35 (31–42) Control group: 43 (36–47) *After education* Intervention group: 25 (22–33) Control group: 42 (36–47) *After treatment* Intervention group: 25 (20–30) Control group: 38 (27–45) *Change in STAI‐S scores with education and the treatment* Intervention group: Median decrease of 10 Control group: Median decrease of 5 *p* value <0.001 STAI‐T *Before education* Intervention group: 37 (34–48) Control group: 45 (38–49) *p* value = 0.102 *After education* Not reported
	Secondary outcome: Change in anxiety scores before information sessions, after information sessions and after treatment (immediately after treatment delivered on the same day as the intervention) Outcome measure: Visual Facial Anxiety Scale (VFAS)^16^	Statistical analysis: chi‐square test VFAS *Before education* Intervention group: 5 (25%) of participants had severe anxiety Control group: 9 (45%) of participants had severe anxiety *p* value = 0.119 *After education* Intervention group: 0 (0%) of participants had severe anxiety Control group: 9 (45%) of participants had severe anxiety after education *p* value = <0.001 *After treatment* Intervention: 0 (0%) of participants had severe anxiety Control: 2 (10%) of participants had severe anxiety *p* value = 0.219 *Change in VFAS scores with education and the treatment* Intervention: *p* value = 0.007 Control: *p* value = 0.001
Koth^21^	Primary outcome: Mean change in anxiety score from preconsultation (before watching the video) to post consultation (after watching the video). Outcome measure: State–Trait Anxiety Inventory (STAI) for adults short form Y‐1^11^, ^12^	Statistical analysis: Paired *t*‐test STAI Y‐1 Intervention: Mean decrease of 1.36 (95% CI ‐3.01‐00.3) Control: Mean decrease of 1.13 (95% CI ‐3.11‐0.85) *p* value = 0.862
Aromatherapy		
Graham^22^	Primary outcome: Change in anxiety score from baseline to treatment completion Outcome measure: Hospital Anxiety and Depression Scale (anxiety) (HADSA)^13^	Statistical analysis: Logistic regression (Odds ratio, 95% CI) HADS‐A Essential oil v nonfragrant placebo: 2.6 (1.1–6.1 95% CI) *p* value = 0.03 Fragrant placebo v nonfragrant placebo: 2.8 (1.1–6.7 95% CI) *p* value = 0.03

**TABLE 4:** Summary statistics for the studies with continuous outcomes.

Study	Measure	N	Mean preintervention	Mean postintervention	SD preintervention	N	Mean preintervention	Mean postintervention	SD preintervention
		Treatment group				Control			
Music interventions									
Chen (2013)	STAI‐S	100	42.63	35.44	11	100	42.03	40.99	10.41
Karadag (2019)	HADS‐A	30	7.8	5.2	3.82	30	7.56	8.56	4.26
O'Callaghan (2012)	STAI‐S	48	37	33	13.1	49	37	31	11.6
O'steen (2021)	STAI‐S	51	39.5	31.5	11.5	51	37.7	33.0	11.5
Smith (2001)	STAI‐S	19	39.6	35.7	13.3	23	38.3	37.3	13.3
Video‐based patient education									
Esen (2022)	STAI‐S	20	35	25	8.1	20	43	42	8.1
Koth (2021)	STAI‐S	39	20.26	18.9	6.41	39	20.44	19.31	8.0
Aromatherapy									
Graham (2003) Essential Oil	HADS‐A	‐	NA	NA	NA	‐	NA	NA	
Graham (2003) Fragrant Placebo	HADS‐A	‐	NA	NA	NA	‐	NA	NA	

*Note*: Participant numbers for O'Callaghan (2012) are lower than reported in Table 1 as three patients withdrew prior to initial radiation therapy. Graham (2003) included 313 patients but did not report how many were randomised to each group.

Abbreviations: CI, confidence interval; HADS‐A, Hospital Anxiety and Depression Scale‐Anxiety; SMD, standardised mean difference; STAI‐S, Spielberger State–Trait Anxiety Inventory.

Anxiety was primarily assessed using the STAI,^42^, ^55^, ^58^ with seven studies including the STAI as a primary outcome measure, six of which used the full measure including both the state and trait subscales,^14^, ^16^, ^17^, ^18^, ^19^, ^20^ and one used the 10 item STAI adult short form Y‐1.^21^ Two studies^15^, ^22^ used the anxiety subscale of the HADS‐A,^43^ one study^20^ used the Visual Facial Anxiety Scale (VFAS)^59^ and one study^14^ reported physiological measures of anxiety, such as blood pressure, heart rate, respiratory rate and fingertip oxygen saturation.^14^

In studies that used outcome measures which were not validated anxiety scales, two different measures were used: one study^17^ used the Symptom Distress Thermometer (SDT),^60^ and one study^18^ used the Psychological Distress Inventory (PDI),^61^ both secondary outcome measures to another anxiety measure.

### 3.2 TIDieR checklist

Reporting according to the TIDieR checklist (Template for Intervention Description and Replication)^62^ for each of the included studies is reported in Table 5.

**TABLE 5:** The TIDieR (Template for Intervention Description and Replication) Checklist.

	Brief name	Why?	What	Who provided	How	Where	When and how much	Tailoring	Modifications	How well: planned?	How well: actual?
Music interventions											
Chen^14^	Pg. 437	Pg. 436	Pg. 437	Pg. 436	Pg. 437	Pg. 437	Pg. 437	Pg. 437	NR	NR	NR
Karadag^15^	Pg. 39	Pg. 40	Pg. 41	NR	Pg. 41	Pg. 41	Pg. 41	NR	NR	Pg. 2373	NR
O'Callaghan^16^	Pg. 473	Pg. 473–474	Pg. 474	NR	Pg. 474	Pg. 474	Pg. 474	Pg. 474	NR	NR	NR
O'Steen^17^	Pg. 670	Pg. 671	Pg. 671	NR	Pg. 671	Pg. 671	Pg. 671	Pg. 671	NR	NR	NR
Raglio^18^	Pg. 1	Pg. 2	Pg. 3	NR	Pg. 3	Pg. 3	Pg. 3	NR	NR	NR	NR
Smith^19^	Pg. 857	Pg. 856	Pg. 857	NR	Pg. 857	Pg. 856	Pg. 857	Pg. 857	NR	NR	NR
Video‐based patient education											
Esen^20^	Pg. 1	Pg. 1	Pg. 2	Pg. 2	Pg. 2	NR	Pg. 2	NR	NR	NR	NR
Koth^21^	Pg. 153	Pg. 154	Pg. 154	Pg. 154	Pg. 154–5	NR	Pg. 154	NR	NR	NR	NR
Aromatherapy											
Graham^22^	Pg. 2372	Pg. 2372	Pg. 2373	Pg. 2373	Pg. 2373	NR	Pg. 2373	NR	Pg. 2372	NR	NR

Abbreviation: NR, not reported.

### 3.3 Methodological quality assessment and risk of bias

Individual ratings and the global rating of methodological quality and assessment of risk of bias are reported in Table 6. All of the studies received a strong rating for study design (either a randomised controlled trial or controlled clinical trial design), data collection methods and withdrawals and drop‐outs. Selection bias was a concern for all nine studies, primarily due to lack of information about recruitment methods. For five of the studies, it was unclear whether confounders were controlled for in the design or the analyses. Finally, for eight of the nine studies it was unclear whether adequate blinding was utilised in the study design. Overall, three studies received a global rating of strong,^14^, ^15^, ^19^ two received a rating of moderate^20^, ^21^ and the remainder (*n* = 4) received a rating of weak.^16^, ^17^, ^18^, ^22^

**TABLE 6:** Methodological quality and risk of bias assessment ratings.

	Selection Bias	Study design	Confounders	Blinding	Data collection methods	Withdrawals and drop‐outs	Overall quality
Music interventions							
Chen^14^	+	+ +	+ +	+	+ +	+ +	+ +
Karadag^15^	+	+ +	+ +	+	+ +	+ +	*++*
O'Callaghan^16^	−	+ +	+ +	−	+ +	+ +	*−*
O'Steen^17^	−	+ +	−	+	+ +	*NA*	*−*
Raglio^18^	−	+ +	−	+	+ +	+ +	*−*
Smith^19^	+	+ +	+ +	+	+ +	+ +	*+ +*
Video‐based patient education							
Esen^20^	+	+ +	−	+	+ +	*NA*	+
Koth^21^	+	+ +	−	+	+ +	*NA*	+
Aromatherapy							
Graham^22^	−	+ +	−	+ +	+ +	+ +	*−*

*Note*: Strong = ‘+ +’. Moderate = ‘+’. Weak = ‘‐‘.

### 3.4 Quality of evidence

Using the GRADE approach^63^ to assess the quality of the evidence, the overall rating of evidence included in this review was deemed very low. The primary outcomes were initially downgraded from high to moderate to reflect the high risk of bias. Specific issues included the overall lack of blinding in the studies, with only one study (of aromatherapy) reporting double blinding of participants and outcome assessors.^22^ The evidence was downgraded another level (from moderate to low) due to heterogeneity of interventions and outcomes. While most of the included studies were music interventions, the other two interventions were video‐based patient education, and aromatherapy. Additionally, while many of the studies used the same outcome measure, results were inconsistently reported, precluding meaningful comparisons between studies. For example, several studies reported the percentage of participants under or above critical values, while others reported mean differences. Finally, the evidence was downgraded at a final level from low to very low, due to clinical heterogeneity and indirectness. Some studies focused on specific cancer groups (i.e. certain cancer sites, diagnoses or treatment techniques), some studying only females, and others being more widely inclusive.

## 4 DISCUSSION

This review aimed to assess the efficacy of nonpharmacological interventions delivered to adult patients with cancer, just prior to or during RT, in reducing procedural anxiety. As far as we are aware, this is the first systematic review to do so. The review identified nine studies that met the inclusion criteria, of which six reported the results of trials of music,^14^, ^15^, ^16^, ^17^, ^18^, ^19^ two reported on trials of video‐based patient education^20^, ^21^ and one on a trial of aromatherapy.^22^ Only three of the included studies^14^, ^15^, ^20^ reported a significant reduction in the primary outcome of self‐reported procedural anxiety for intervention participants, two music interventions^14^, ^15^ and one video‐based patient education.^20^ Of these, the two music intervention studies received a methodological quality rating of strong,^14^, ^15^ and the video‐based patient education study received a moderate rating.^20^ Only one study, a music intervention,^14^ reported a significant reduction in the secondary outcome of physiological symptoms of procedural anxiety (systolic blood pressure).

This review identified some promise of music interventions in reducing procedural anxiety in patients undergoing RT. Of the two studies that reported significant findings,^14^, ^15^ both received strong methodological quality and low risk of bias ratings. In addition to statistically significant reductions in anxiety, both interventions also reported clinically meaningfully reductions in mean anxiety scores. The two interventions were both delivered via headphones; however, they varied in terms of where (treatment bunker^15^ vs. waiting room^14^), when (during treatment^15^ vs. prior to treatment^14^) and how often the intervention was delivered (over many sessions^15^ vs. prior to a single treatment session^14^) and whether the music was self‐selected^14^ or not.^15^ It is possible that the collection of songs in the study conducted by Chen and colleagues^14^ shared similarities to the piece of music chosen for the Karadag and colleagues’^15^ study; however, the authors are not qualified to comment on the therapeutic benefit of particular song choices. Finally, inclusion criteria in the two studies differed, with Chen and colleagues^14^ including all cancer sites, while Karadag and colleagues^15^ included only patients with early‐stage breast cancer (thus limiting the population by cancer site and also gender).

The remaining studies of music reported no significant effect on procedural anxiety. Like the two aforementioned studies, the delivery of these interventions varied, with no clear differences from those that reported significant findings. However, three^16^, ^17^, ^18^ of the four studies^16^, ^17^, ^18^, ^19^ reporting nonsignificant findings received an overall quality and risk of bias rating of ‘weak’. This suggests that further, high‐quality research may be warranted to rigorously explore the effect of music listening on procedural anxiety, and identify the important factors that contribute to a therapeutic effect of music.

This review identified equivocal findings in the studies trialling video‐based patient education. While no effect was reported in the study conducted by Koth and colleagues,^21^ a significant reduction in procedural anxiety was reported in the study conducted by Esen and colleagues.^20^ Overall, both video‐based patient education studies received a moderate rating for methodological quality and risk of bias, with identical scores on each of the domains. However, the significant findings reported in the Esen study should be interpreted with a reasonable amount of caution. The intervention and control groups in this study differed considerably in preintervention anxiety scores, with the mean anxiety score for the control group exceeding the cut off for clinically significant anxiety (**≥** 40). Therefore, it is entirely possible that the intervention may have been more effective for those experiencing anxiety in the nonclinical range, resulting in a greater reduction in the outcome measure for the intervention group.

This review identified only one trial of aromatherapy,^22^ that reported no effect of aromatherapy on anxiety. While the trial comprised a large sample size, it received a global rating of ‘weak’ on the methodological quality and risk of bias tool, due to selection bias and confounders. Therefore, some further exploration of aromatherapy may be warranted.

It is worth noting some limitations of the interventions included in this review.

Overall, none of the interventions screened participants for clinically significant anxiety as part of eligibility for study participation. It is possible that samples with preintervention levels of anxiety in the normal range could prevent a measurable reduction in anxiety. Additionally, the study by Karadag and colleagues excluded women with left‐sided breast cancer in this study. This criterion was due to the RT treatment protocol for left‐sided cancers, which involves a breath‐holding technique, precluding the use of a music listening during the treatment delivery. As this intervention was only trialled on women with breast cancer, the exclusion of women with left‐sided cancers is quite a significant limitation. While the delivery of the intervention inside the treatment bunker produced a statistically significant and clinically meaningful reduction in anxiety, the suitability of this intervention is dependent on the RT treatment protocol (e.g. precluding those having treatment for left‐sided breast cancer) and therefore limits the clinical utility of this intervention.

There is considerable scope for further innovative research in this area, given the prevalence and impact of procedure related anxiety in the RT setting. Further examination of music interventions and video‐based patient education is warranted using large, rigorous trials. It is worth noting the clinical heterogeneity among the trials included in this review, between those that reported an effect of their intervention and those that did not. It is possible that some interventions may be more suited to certain populations. Future research should endeavour to identify the key elements of these interventions for different clinical populations. Additionally, future trials should be designed to only provide interventions to patients who report raised anxiety levels. If patients do not report anxiety at baseline there is no scope for an intervention to reduce anxiety scores (floor effects) and this may obscure any beneficial effects of an intervention.

Another useful avenue for further research would be a review of psychological interventions to complement the current review. A systematic review of the literature of psychological interventions for people with head and neck cancer identified 21 intervention studies between 1980 and 2017.^64^ However none of these targeted procedural anxiety and all but one required multiple sessions.

We note that patients experiencing clinically severe anxiety, for example a specific phobia which is inhibiting their ability to undergo treatment, are likely to require additional specialised assistance from a trained psychologist. Case studies of cognitive behavioural therapy including graded exposure therapy/systematic desensitisation offer a promising avenue in this regard.^65^, ^66^

### 4.1 Review limitations

There was a high degree of heterogeneity between studies and variability in validated tools used, limiting our ability to confidently pool any data. The inconsistency in findings, and the overall very low rating of the evidence precludes any firm conclusions. A key limitation of this review is that it is possible some of the interventions included in the review may be treating generalised anxiety. The term ‘procedural anxiety’ is not used consistently, so we chose to focus on studies that target anxiety with interventions just prior to or during RT, using standardised measures typically used to measure procedural anxiety.^23^, ^67^, ^68^, ^69^

### 4.2 Clinical implications

Overall, there is no clear evidence base on which to guide clinical practice. There are very few well‐designed studies of promising interventions, precluding any specific recommendations regarding interventions to reduce procedural anxiety in the RT setting.

## 5 CONCLUSION

These findings highlight the paucity of good quality evidence regarding nonpharmacological interventions targeting procedural anxiety in the RT setting. While there is limited evidence of promise in music and education‐based interventions, it is unclear which elements of these interventions contributed to a reduction in procedural anxiety. There is significant scope for improvement in this area, especially improved methodological quality and reduced heterogeneity. Future research should not only seek to determine which key elements of interventions are important for a therapeutic effect in defined clinical populations, but also explore novel interventions that have shown promise in reducing anxiety in other medical settings.

## AUTHOR CONTRIBUTIONS

**Erin Forbes:** Conceptualization (lead); formal analysis (lead); methodology (lead); project administration (lead); writing – original draft (lead); writing – review and editing (equal). **Amanda L Baker:** Conceptualization (equal); methodology (equal); supervision (equal); visualization (equal); writing – review and editing (equal). **Ben Britton:** Conceptualization (equal); methodology (equal); supervision (equal); visualization (equal); writing – review and editing (supporting). **Kerrie Clover:** Conceptualization (equal); methodology (equal); supervision (equal); visualization (equal); writing – review and editing (equal). **Eliza Skelton:** Data curation (equal); formal analysis (equal); writing – review and editing (equal). **Lyndell Moore:** Data curation (equal); methodology (equal); writing – review and editing (equal). **Tonelle E. Handley:** Data curation (equal); methodology (equal); writing – review and editing (equal). **Sharon Oultram:** Investigation (equal); methodology (equal); writing – review and editing (equal). **Christopher Oldmeadow:** Formal analysis (equal); writing – review and editing (equal). **Alison Gibberd:** Formal analysis (equal); investigation (equal); writing – review and editing (equal). **Kristen McCarter:** Conceptualization (equal); methodology (equal); supervision (lead); visualization (equal); writing – review and editing (equal).

## CONFLICT OF INTEREST STATEMENT

The authors have no conflicts of interest to declare.

## Supporting information


Appendix S1

## Data Availability

NA
