# Supplementary material for: A systematic review of nonpharmacological interventions to reduce procedural anxiety among patients undergoing radiation therapy for cancer
Source: Cancer Med. 2023 Oct 6;12(20):20396–422. doi: 10.1002/cam4.6573 (PMC10652309; doi:10.1002/cam4.6573)
Supplement: Supplementary file 1 — Appendix S1 [file CAM4-12-20396-s001.pdf]

APPENDIX 1: MEDLINE SEARCH STRATEGY

1	RADIOTHERAPY/
2	radiation therapy.mp.
3	Radiation Oncology/
4	(radiotherapy or radiation oncology).tw.
5	1 or 2 or 3 or 4
6	anxiety/ or fear/ or panic/
7	Stress, Psychological/
8	distress.mp.
9	Depression/
10	Stress Disorders, Traumatic, Acute/
11	anxiety disorders/ or panic disorder/ or phobic disorders/
12	"Quality of Life"/
13	(anxiety or anxious or fear* or panic* or depress*).tw.
14	6 or 7 or 8 or 9 or 10 or 11 or 12 or 13
15	exp Mind-Body Therapies/
16	Music Therapy/
17	psychotherapy/ or aromatherapy/ or feedback, psychological/
18	Patient Education as Topic/
19	BIBLIOTHERAPY/
20	Desensitization, Psychologic/
21	(mindfulness or relaxation or psychotherapy or mind body therap* or yoga or aromatherapy or breathing exercis* or music therap* or bibliotherap* or meditation* or biofeedback or distraction).tw.

22 15 or 16 or 17 or 18 or 19 or 20 or 21

23 5 and 14 and 22
